# Redetermination of olivenite from an untwinned single-crystal

**DOI:** 10.1107/S1600536808026676

**Published:** 2008-08-23

**Authors:** Chen Li, Hexiong Yang, Robert T. Downs

**Affiliations:** a Department of Geosciences, University of Arizona, 1040 E. 4th Street, Tucson, AZ 85721-0077, USA

## Abstract

The crystal structure of olivenite, ideally Cu_2_(AsO_4_)(OH) [dicopper(II) arsenate(V) hydroxide], was redetermined from an untwinned and phosphate-containing natural sample, composition Cu_2_(As_0.92_P_0.08_O_4_), from Majuba Hill (Nevada, USA). Olivenite is structurally analogous with the important rock-forming mineral andalusite, Al_2_OSiO_4_. Its structure consists of chains of edge-sharing, distorted [CuO_4_(OH)_2_] octa­hedra extending parallel to [001]. These chains are cross-linked by isolated AsO_4_ tetra­hedra through corner-sharing, forming channels in which dimers of edge-sharing [CuO_4_(OH)] trigonal bipyramids are located. The structure is stabilized by medium to weak O—H⋯O hydrogen bonds. In contrast to the previous refinements from powder and single crystal X-ray data, all non-H atoms were refined with anisotropic displacement parameters and the H atom was located.

## Related literature

For olivenite, see: Heritsch (1938 ▶); Richmond (1940 ▶); Berry (1951 ▶); Walitzi (1963 ▶); Toman (1977 ▶); Burns & Hawthorne (1995 ▶). For other minerals of the olivenite group, see: Hill (1976 ▶); Cordsen (1978 ▶); Frost *et al.* (2002 ▶). For correlations between O—H stretching frequencies and O—H⋯O donor–acceptor distances, see: Libowitzky (1999 ▶). For general background, see: Robinson *et al.* (1971 ▶).

## Experimental

### 

#### Crystal data

Cu_2_[(As_0.92_·P_0.08_)O_4_]OH
*M* *_r_* = 278.61
Monoclinic, 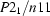

*a* = 8.5844 (3) Å
*b* = 8.2084 (3) Å
*c* = 5.9258 (2) Å
α = 90.130 (2)°
*V* = 417.56 (3) Å^3^
*Z* = 4
Mo *K*α radiation
μ = 17.32 mm^−1^
*T* = 293 (2) K
0.06 × 0.05 × 0.05 mm

#### Data collection

Bruker APEX2 CCD area-detector diffractometer
Absorption correction: multi-scan (*SADABS*; Sheldrick, 2005 ▶) *T*_min_ = 0.425, *T*_max_ = 0.480 (expected range = 0.372–0.421)
7604 measured reflections
1580 independent reflections
1372 reflections with *I* > 2σ(*I*)
*R*_int_ = 0.029

#### Refinement

*R*[*F* ^2^ > 2σ(*F* ^2^)] = 0.020
*wR*(*F* ^2^) = 0.045
*S* = 1.10
1580 reflections
80 parameters
All H-atom parameters refined
Δρ_max_ = 0.55 e Å^−3^
Δρ_min_ = −0.84 e Å^−3^

### 

Data collection: *APEX2* (Bruker, 2003 ▶); cell refinement: *SAINT* (Bruker, 2005 ▶); data reduction: *SAINT*; program(s) used to solve structure: *SHELXS97* (Sheldrick, 2008 ▶); program(s) used to refine structure: *SHELXL97* (Sheldrick, 2008 ▶); molecular graphics: *XtalDraw* (Downs & Hall-Wallace, 2003 ▶); software used to prepare material for publication: *SHELXTL* (Sheldrick, 2008 ▶).

## Supplementary Material

Crystal structure: contains datablocks I, New_Global_Publ_Block. DOI: 10.1107/S1600536808026676/wm2187sup1.cif

Structure factors: contains datablocks I. DOI: 10.1107/S1600536808026676/wm2187Isup2.hkl

Additional supplementary materials:  crystallographic information; 3D view; checkCIF report

## Figures and Tables

**Table 1:** Selected bond lengths (Å)

As1—O4	1.6479 (18)
As1—O5	1.6644 (19)
As1—O1	1.6855 (17)
As1—O2	1.6866 (16)
Cu1—O1^i^	1.9466 (18)
Cu1—O3*H*	1.9546 (18)
Cu1—O5^ii^	1.9940 (18)
Cu1—O1^iii^	2.0132 (17)
Cu1—O4	2.1418 (18)
Cu2—O2	1.9526 (18)
Cu2—O2^iv^	1.9715 (17)
Cu2—O3*H*^v^	1.9913 (18)
Cu2—O3*H*	2.0001 (17)
Cu2—O5^vi^	2.3439 (17)
Cu2—O4^iii^	2.3874 (18)

Symmetry codes: (i) 
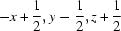
; (ii) 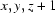; (iii) 
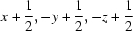
; (iv) 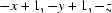; (v) 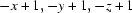; (vi) 
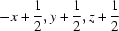
.

**Table 2:** Hydrogen-bond geometry (Å, °)

*D*—H⋯*A*	*D*—H	H⋯*A*	*D*⋯*A*	*D*—H⋯*A*
O3*H*—H1⋯O4^vi^	0.67 (4)	2.35 (4)	2.788 (3)	125 (4)
O3*H*—H1⋯O5^vi^	0.67 (4)	2.66 (4)	2.977 (2)	112 (4)

Symmetry code: (vi) 
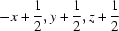
.

**Fig. 1.**
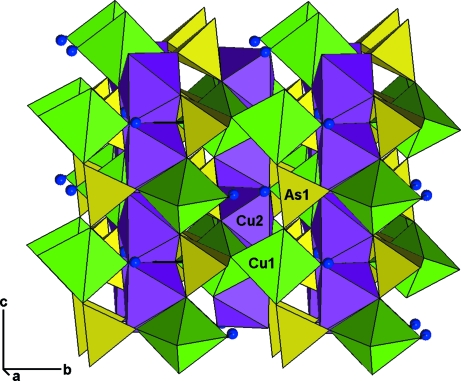
The crystal structure of olivenite given in the polyhedral representation. The H atoms (blue spheres) are drawn with an arbitrary radius.

## supplementary crystallographic information

### Comment

Olivenite, ideally Cu_2_(AsO_4_)(OH), is a common secondary mineral of the oxidized zone of hydrothermal deposits. It crystallizes with monoclinic symmetry in space group *P*2_1_/*n* with a pseudo-orthorhombic cell (β~ 90°). Several arsenates and phosphates belong to the olivenite mineral group, including adamite, Zn_2_(AsO_4_)(OH), eveite, Mn^2+^_2_(AsO_4_)(OH), libethenite, Cu_2_(PO_4_)(OH), zincolibethenite, CuZn(PO_4_)OH, and zincolivenite, CuZn(AsO_4_)(OH). Interestingly, except olivenite, all other minerals in this group display orthorhombic symmetry and crystallize in space group *Pnnm*. The first approximate structure determination of olivenite was reported by Heritsch (1938) in space group *Pnnm*. Subsequent studies on olivenite, however, proposed various other space groups: *P*2_1_2_1_2_1_ (Richmond, 1940), *Pnmm* (Berry, 1951), and *Pn*2_1_*m *(Walitzi, 1963). Toman (1977) proposed that olivenite has actually monoclinic symmetry, and that most of the crystals are twinned. Structure refinements based on single-crystal X-ray diffraction data, uncorrected and corrected for twinning, yielded reliability factors *R*(F) of 0.090 and 0.065, respectively. However, Toman (1977) did not report any atomic displacement parameters or the position of the H atom. To avoid the complication of interpreting X-ray diffraction intensity data due to twinning, Burns & Hawthorne (1995) performed structure refinements of olivenite using the Rietveld method from powder X-ray diffraction data. By assuming a single isotropic displacement parameter for all O atoms and no H atom position, they attempted refinements both in space group *Pnnm* and *P*2_1_/*n* and obtained nearly identical *R*_Bragg_ factors (~ 0.074) and goodness-of-fit values (~ 2.30). In our efforts to understand the relationships between the hydrogen environments and Raman spectra of hydrous minerals, we concluded that the structural information of olivenite needs to be improved. During the course of sample identification for the RRUFF project, we discovered an untwinned and phosphate-containing single-crystal of olivenite from Majuba Hill, Pershing County, Nevada, USA, and conducted a detailed structure refinement.

The structure of olivenite consists of chains of edge-sharing [Cu2O_4_(OH)_2_] octahedra extending parallel to [001] that are cross-linked by sharing corners with isolated AsO_4_ tetrahedra to form an open framework. Channels in the framework contain dimers of edge-sharing [Cu1O_4_(OH)] trigonal bipyramids that share corners with the [Cu2O_4_(OH)_2_] octahedra and AsO_4_ tetrahedra (Fig. 1). Although the average <As1—O>, <Cu1—O>, and <Cu2—O> distances of our study agree well with those given by Toman (1977) and Burns & Hawthorne (1995), the corresponding individual bond distances and angles from the three structure refinements (including ours) vary significantly. For example, the shortest and longest As—O bond distances within the AsO_4_ tetrahedra are 1.618 Å (As—O5) and 1.731 Å (As—O4), respectively, from Toman (1977), 1.640 Å (As—O4) and 1.702 Å (As—O1) from Burns & Hawthorne (1995), and 1.6479 (18) Å (As—O4) and 1.6866 (16) Å (As—O2) from this study. The shortest Cu—O bond length within the [Cu1O_4_(OH)] trigonal bipyramid is 1.9466 (18) Å (Cu1—O1) from this study, but is 1.917 Å (Cu1—O3) from Toman (1977) and 1.938 Å (Cu1—O5) from Burns & Hawthorne (1995). Furthermore, the AsO_4_ tetrahedron reported by Burns & Hawthorne (1995) is remarkably distorted, as measured by the tetrahedral angle variance (TAV) and quadratic elongation (TQE) (Robinson *et al.*, 1971), which are 105.3 and 1.0281, respectively. In comparison, the TAV and TQE values are 6.1 and 1.0027, respectively, from Toman (1977), and 2.8 and 1.0008 from this study.

The H atom is bonded to O3, at a separation of 0.67 (4) Å. This distance is in fairly agreement with that (0.77 Å) reported for adamite (Hill, 1976). Our Raman spectra of olivenite (*http://rruff.info/olivenite/R040181*) show two major bands in the hydroxyl stretching (ν_OH_) region: one at 3440 cm^-1^ and the other at 3464 cm^-1^. Similar wavenumbers (ν_OH_= 3437 and 3464 cm^-1^) were also obtained by Frost *et al.* (2002). Given the correlation between ν_OH_ and O—H···O distances in minerals (Libowitzky, 1999), one would expect two O—H···O distances between 2.8 and 2.9 Å in olivenite. Our structural data indeed show that the O3(=OH) atom is at a distance of 2.79 Å from O4 and 2.98 Å from O5. Nevertheless, the angles O3—H···O4 (125°) and O3—H···O5 (112°) appear to be too small for hydrogen bonding. Note that, based on the structure refinement of libethenite, the phosphate analogue of olivenite, Cordsen (1978) proposed a bifurcated hydrogen bonding model for this mineral, in which there are two O—H···O bonds at the same distance of 2.84 Å and two bonding angles of 110°.

### Experimental

The olivenite crystal used in this study is from Majuba Hill, Pershing County, Nevada (USA) and is a sample from the RRUFF project (deposition No. R040181; http//rruff.info). The chemical composition, (Cu_0.98_□_0.02_)_2_(As_0.90_P_0.10_O_4_)(OH)_0.92_, was determined with a CAMECA SX50 electron microprobe (http//rruff.info).

### Refinement

The setting in *P*2_1_/*n*11 with the *a*-axis as the monoclinic axis was chosen to keep consistency with previous studies on this mineral (Toman, 1977; Burns & Hawthorne, 1995). The minor vacancies for Cu and OH determined from electron microprobe analysis were ignored during the final refinement and all corresponding sites were assumed to be fully occupied. The relative ratio of As versus P at the As1 site was allowed to vary freely, with the sum of site occupation factors constrained to unity. The results agree well with those obtained from electron microprobe analysis. The H atom was located in a difference Fourier map, and its position was refined freely. The highest residual peak in the difference Fourier maps was located 0.80 Å from atom O1, and the deepest hole was located 0.63 Å from Cu1.

### Crystal data

**Table:**

Cu_2_[(As_0.92_·P_0.08_)O_4_]OH	*F*_000_ = 521
*M**_r_* = 278.61	*D*_x_ = 4.444 Mg m^−^^3^
Monoclinic, *P*2_1_/*n*11	Mo *K*α radiation λ = 0.71073 Å
Hall symbol: -P 2xn	Cell parameters from 1588 reflections
*a* = 8.5844 (3) Å	θ = 3.9–30.1º
*b* = 8.2084 (3) Å	µ = 17.32 mm^−^^1^
*c* = 5.9258 (2) Å	*T* = 293 (2) K
β = 90º	Euhedral, equant, green
*V* = 417.56 (3) Å^3^	0.06 × 0.05 × 0.05 mm
*Z* = 4	

### Data collection

**Table:**

Bruker APEX2 CCD area-detector diffractometer	1580 independent reflections
Radiation source: fine-focus sealed tube	1372 reflections with *I* > 2σ(*I*)
Monochromator: graphite	*R*_int_ = 0.029
*T* = 293(2) K	θ_max_ = 33.1º
φ and ω–scans	θ_min_ = 3.4º
Absorption correction: multi-scan(SADABS; Sheldrick, 2005)	*h* = −12→13
*T*_min_ = 0.425, *T*_max_ = 0.480	*k* = −12→12
7604 measured reflections	*l* = −9→9

### Refinement

**Table:**

Refinement on *F*^2^	Secondary atom site location: difference Fourier map
Least-squares matrix: full	*w* = 1/[σ^2^(*F*_o_^2^) + (0.0158*P*)^2^ + 0.4808*P*] where *P* = (*F*_o_^2^ + 2*F*_c_^2^)/3
*R*[*F*^2^ > 2σ(*F*^2^)] = 0.020	(Δ/σ)_max_ = 0.001
*wR*(*F*^2^) = 0.046	Δρ_max_ = 0.55 e Å^−^^3^
*S* = 1.10	Δρ_min_ = −0.84 e Å^−^^3^
1580 reflections	Extinction correction: SHELXL, Fc^*^=kFc[1+0.001xFc^2^λ^3^/sin(2θ)]^-1/4^
80 parameters	Extinction coefficient: 0.0009 (4)
Primary atom site location: structure-invariant direct methods	

### Special details

**Table:**

Geometry. All e.s.d.'s (except the e.s.d. in the dihedral angle between two l.s. planes) are estimated using the full covariance matrix. The cell e.s.d.'s are taken into account individually in the estimation of e.s.d.'s in distances, angles and torsion angles; correlations between e.s.d.'s in cell parameters are only used when they are defined by crystal symmetry. An approximate (isotropic) treatment of cell e.s.d.'s is used for estimating e.s.d.'s involving l.s. planes.
Refinement. Refinement of *F*^2^ against ALL reflections. The weighted *R*-factor *wR* and goodness of fit *S* are based on *F*^2^, conventional *R*-factors *R* are based on *F*, with *F* set to zero for negative *F*^2^. The threshold expression of *F*^2^ > σ(*F*^2^) is used only for calculating *R*-factors(gt) *etc*. and is not relevant to the choice of reflections for refinement. *R*-factors based on *F*^2^ are statistically about twice as large as those based on *F*, and *R*- factors based on ALL data will be even larger.

### Fractional atomic coordinates and isotropic or equivalent isotropic displacement parameters (Å^2^)

**Table:**

	*x*	*y*	*z*	*U*_iso_*/*U*_eq_	Occ. (<1)
As1	0.24984 (3)	0.26286 (3)	0.01069 (4)	0.00764 (8)	0.916 (3)
P1	0.24984 (3)	0.26286 (3)	0.01069 (4)	0.00764 (8)	0.084 (3)
Cu1	0.38101 (3)	0.13699 (3)	0.52383 (5)	0.01125 (8)	
Cu2	0.49966 (4)	0.50071 (3)	0.24933 (5)	0.01022 (8)	
O1	0.1070 (2)	0.4010 (2)	0.0500 (4)	0.0148 (4)	
O2	0.41871 (19)	0.3677 (2)	0.0022 (3)	0.0103 (3)	
O3H	0.4028 (2)	0.3734 (2)	0.5002 (3)	0.0095 (3)	
O4	0.2470 (2)	0.1303 (2)	0.2191 (3)	0.0144 (4)	
O5	0.2226 (2)	0.1661 (2)	−0.2333 (3)	0.0144 (4)	
H1	0.329 (4)	0.400 (5)	0.510 (7)	0.023 (10)*	

### Atomic displacement parameters (Å^2^)

**Table:**

	*U*^11^	*U*^22^	*U*^33^	*U*^12^	*U*^13^	*U*^23^
As1	0.00795 (12)	0.00743 (12)	0.00753 (12)	−0.00088 (8)	−0.00011 (8)	−0.00007 (8)
P1	0.00795 (12)	0.00743 (12)	0.00753 (12)	−0.00088 (8)	−0.00011 (8)	−0.00007 (8)
Cu1	0.01148 (14)	0.00725 (13)	0.01503 (15)	−0.00026 (10)	0.00225 (11)	−0.00003 (10)
Cu2	0.01370 (14)	0.01085 (14)	0.00612 (13)	−0.00421 (10)	−0.00003 (10)	−0.00002 (10)
O1	0.0108 (8)	0.0081 (7)	0.0255 (10)	0.0001 (6)	−0.0006 (7)	−0.0003 (7)
O2	0.0114 (7)	0.0118 (8)	0.0077 (8)	−0.0054 (6)	−0.0002 (6)	0.0010 (6)
O3H	0.0101 (8)	0.0084 (7)	0.0100 (8)	0.0000 (6)	0.0002 (6)	0.0005 (6)
O4	0.0164 (8)	0.0154 (8)	0.0114 (8)	−0.0059 (7)	−0.0036 (6)	0.0032 (7)
O5	0.0142 (8)	0.0178 (9)	0.0112 (8)	−0.0047 (6)	0.0022 (6)	−0.0035 (7)

### Geometric parameters (Å, °)

**Table:**

As1—O4	1.6479 (18)	Cu2—O2	1.9526 (18)
As1—O5	1.6644 (19)	Cu2—O2^v^	1.9715 (17)
As1—O1	1.6855 (17)	Cu2—O3H^vi^	1.9913 (18)
As1—O2	1.6866 (16)	Cu2—O3H	2.0001 (17)
Cu1—O1^i^	1.9466 (18)	Cu2—O5^vii^	2.3439 (17)
Cu1—O3H	1.9546 (18)	Cu2—O4^iii^	2.3874 (18)
Cu1—O5^ii^	1.9940 (18)	Cu2—Cu2^v^	2.9549 (6)
Cu1—O1^iii^	2.0132 (17)	Cu2—Cu2^vi^	2.9710 (6)
Cu1—O4	2.1418 (18)	O3H—H1	0.67 (4)
Cu1—Cu1^iv^	3.0509 (6)		
			
O4—As1—O5	109.53 (9)	O3H—Cu2—O5^vii^	86.14 (7)
O4—As1—O1	109.30 (10)	O2—Cu2—O4^iii^	97.12 (7)
O5—As1—O1	109.72 (9)	O2^v^—Cu2—O4^iii^	89.48 (7)
O4—As1—O2	111.90 (8)	O3H^vi^—Cu2—O4^iii^	78.52 (7)
O5—As1—O2	109.70 (9)	O3H—Cu2—O4^iii^	94.30 (7)
O1—As1—O2	106.63 (9)	O5^vii^—Cu2—O4^iii^	168.95 (7)
O1^i^—Cu1—O3H	171.47 (7)	As1—O1—Cu1^viii^	128.31 (10)
O1^i^—Cu1—O5^ii^	95.51 (8)	As1—O1—Cu1^ix^	124.56 (10)
O3H—Cu1—O5^ii^	89.99 (8)	Cu1^viii^—O1—Cu1^ix^	100.78 (8)
O1^i^—Cu1—O1^iii^	79.22 (8)	As1—O2—Cu2	124.61 (10)
O3H—Cu1—O1^iii^	92.59 (7)	As1—O2—Cu2^v^	127.49 (10)
O5^ii^—Cu1—O1^iii^	146.34 (8)	Cu2—O2—Cu2^v^	97.70 (7)
O1^i^—Cu1—O4	94.12 (8)	Cu1—O3H—Cu2^vi^	120.04 (9)
O3H—Cu1—O4	90.83 (7)	Cu1—O3H—Cu2	127.85 (10)
O5^ii^—Cu1—O4	104.18 (8)	Cu2^vi^—O3H—Cu2	96.20 (7)
O1^iii^—Cu1—O4	109.33 (8)	Cu1—O3H—H1	103 (3)
O2—Cu2—O2^v^	82.30 (7)	Cu2^vi^—O3H—H1	99 (3)
O2—Cu2—O3H^vi^	175.64 (7)	Cu2—O3H—H1	107 (3)
O2^v^—Cu2—O3H^vi^	97.45 (7)	As1—O4—Cu1	127.46 (10)
O2—Cu2—O3H	96.73 (7)	As1—O4—Cu2^ix^	111.74 (9)
O2^v^—Cu2—O3H	176.19 (7)	Cu1—O4—Cu2^ix^	114.99 (8)
O3H^vi^—Cu2—O3H	83.80 (7)	As1—O5—Cu1^x^	125.98 (10)
O2—Cu2—O5^vii^	93.79 (7)	As1—O5—Cu2^xi^	115.32 (9)
O2^v^—Cu2—O5^vii^	90.25 (7)	Cu1^x^—O5—Cu2^xi^	117.06 (9)
O3H^vi^—Cu2—O5^vii^	90.56 (7)		

Symmetry codes: (i) −*x*+1/2, *y*−1/2, *z*+1/2; (ii) *x*, *y*, *z*+1; (iii) *x*+1/2, −*y*+1/2, −*z*+1/2; (iv) −*x*+1, −*y*, −*z*+1; (v) −*x*+1, −*y*+1, −*z*; (vi) −*x*+1, −*y*+1, −*z*+1; (vii) −*x*+1/2, *y*+1/2, *z*+1/2; (viii) −*x*+1/2, *y*+1/2, *z*−1/2; (ix) *x*−1/2, −*y*+1/2, −*z*+1/2; (x) *x*, *y*, *z*−1; (xi) −*x*+1/2, *y*−1/2, *z*−1/2.

### Hydrogen-bond geometry (Å, °)

**Table:**

*D*—H···*A*	*D*—H	H···*A*	*D*···*A*	*D*—H···*A*
O3H—H1···O4^vii^	0.67 (4)	2.35 (4)	2.788 (3)	125 (4)
O3H—H1···O5^vii^	0.67 (4)	2.66 (4)	2.977 (2)	112 (4)

Symmetry codes: (vii) −*x*+1/2, *y*+1/2, *z*+1/2.
